# Biological Differences between Brackish and Fresh Water-Derived *Aedes aegypti* from Two Locations in the Jaffna Peninsula of Sri Lanka and the Implications for Arboviral Disease Transmission

**DOI:** 10.1371/journal.pone.0104977

**Published:** 2014-08-29

**Authors:** Ranjan Ramasamy, Pavilupillai J. Jude, Thabothiny Veluppillai, Thampoe Eswaramohan, Sinnathamby N. Surendran

**Affiliations:** Department of Zoology, Faculty of Science, University of Jaffna, Jaffna, Sri Lanka; New Mexico State University, United States of America

## Abstract

The mainly fresh water arboviral vector *Aedes aegypti* L. (Diptera: Culicidae) can also undergo pre-imaginal development in brackish water of up to 15 ppt (parts per thousand) salt in coastal areas. We investigated differences in salinity tolerance, egg laying preference, egg hatching and larval development times and resistance to common insecticides in *Ae. aegypti* collected from brackish and fresh water habitats in Jaffna, Sri Lanka. Brackish water-derived *Ae. aegypti* were more tolerant of salinity than fresh water-derived *Ae. aegypti* and this difference was only partly reduced after their transfer to fresh water for up to five generations. Brackish water-derived *Ae. aegypti* did not significantly discriminate between 10 ppt salt brackish water and fresh water for oviposition, while fresh water-derived *Ae. aegypti* preferred fresh water. The hatching of eggs from both brackish and fresh water-derived *Ae. aegypti* was less efficient and the time taken for larvae to develop into pupae was prolonged in 10 ppt salt brackish water. *Ae. aegypti* isolated from coastal brackish water were less resistant to the organophosphate insecticide malathion than inland fresh water *Ae. aegypti*. Brackish and fresh water-derived *Ae. aegypti* however were able to mate and produce viable offspring in the laboratory. The results suggest that development in brackish water is characterised by pertinent biological changes, and that there is restricted genetic exchange between coastal brackish and inland fresh water *Ae. aegypti* isolates from sites 5 km apart. The findings highlight the need for monitoring *Ae. aegypti* developing in coastal brackish waters and extending vector control measures to their habitats.

## Introduction

*Aedes aegypti* L. (Diptera: Culicidae) is the principal mosquito vector of yellow fever and, together with the closely related *Aedes albopictus* Skuse, a primary vector of dengue and chikungunya [1]–[6]. Dengue is the most common arboviral disease of humans, with 50 million annual cases in more than 100 countries, an increasing incidence and global spread, and a 2.5% fatality rate in severe dengue [4], [5]. There is presently no licensed vaccine or specific anti-viral drug for dengue [5]. Yellow fever has a zoonotic reservoir, is endemic to Africa and South America with the potential to spread to Asia and, although an effective live-attenuated vaccine is available, responsible for 200,000 cases and 30,000 deaths in the world every year [6]. Chikungunya, a debilitating but not often fatal arboviral disease, occurs in Asia, Africa and the Americas, and caused a recent epidemic in temperate Europe [3]. A vaccine against chikungunya is not yet available. Dengue and chikungunya are endemic in Sri Lanka with a high incidence in the northern Jaffna peninsula [7], [8].

The control of dengue and chikungunya therefore relies heavily on surveillance for *Ae. aegypti* and *Ae. albopictus* and larviciding and eliminating pre-imaginal development habitats [3], [5]. The two vectors have been long regarded to lay eggs and undergo pre-imaginal development only in fresh water collections (e.g. blocked drains, roof gutters, flower pot bases, leaf axils) near human settlements that are the principal targets for control measures worldwide [2], [5], [9]. Recent data however show that both vectors can also develop in brackish water collections in coastal areas of Sri Lanka and Brunei Darussalam [10]–[14]. Fresh, brackish and saline waters are respectively defined as containing <0.5, 0.5 to 30, and >30 ppt (parts per thousand) salt [10]. Pre-imaginal stages of *Ae. aegypti* and *Ae. albopictus* develop in brackish water in discarded containers and domestic wells of up to 15 ppt and 14 ppt salinity respectively in northern and eastern Sri Lanka [10]–[12]. Salinity tolerance in the two *Aedes* vectors therefore has implications for the effective control of arboviral diseases particularly in a future context of rising sea levels increasing the extent of coastal brackish water habitats [14]–[16].

*Aedes aegypti* larvae from a long-established laboratory colony tolerate a short-term increase in salinity of up to 10 ppt through a reversible osmoconformation mechanism involving the accumulation of amino acids and ions in the haemolymph [17]. *Aedes aegypti* are able to oviposit in brackish water of up to 18 ppt in field conditions [10]. *Aedes aegypti* collected from coastal locations are able to undergo preimaginal development in brackish water in the laboratory [10], [18]. Some other mosquito species have evolved to tolerate salinity through genetic changes. Salinity-tolerant mosquito larvae possess cuticles that are less permeable to water than freshwater forms, and their pupae have thickened and sclerotized cuticles that are impermeable to water and ions [19]. Salinity-tolerant species have also evolved various physiological mechanisms to cope with salinity in the larval environment. *Aedes taeniorhynchus* larvae ingest the surrounding fluid and excrete Na^+^ and Cl^−^ from the posterior rectum to produce a hyperosmotic urine [19]. *Culex tarsalis* larvae accumulate amino acids and trehalose in the hemolymph to maintain iso-osmolarity in brackish waters in an osmoconformation process [20]. Larvae of the malaria vector *Anopheles albimanus* are able to differentially localize sodium-potassium ATPase in rectal cells in fresh or saline water for likely osmoregulation through ion excretion [21]. There is evidence to suggest that a similar adaptation accompanied speciation of salinity-tolerant *Anopheles merus* within the predominantly fresh water *Anopheles gambiae* complex of malaria vectors in Africa [22].

There is presently no information on possible genetic and physiological changes associated with brackish water development in field *Ae. aegypti* populations. This information is relevant for developing more effective measures for controlling dengue, chikungunya and other arboviral diseases. It has been proposed that differences in salinity tolerance between *Ae. aegypti* isolates from northern and eastern Sri Lanka may be due to genetic variation [10].

We hypothesised that *Ae. aegypti* developing in coastal brackish water habitats differ from inland fresh water *Ae. aegypti* in the Jaffna peninsula in salinity tolerance, oviposition preference, egg hatching and larval development times and insecticide resistance. We tested this by measuring these characteristics in the laboratory in *Ae. aegypti* collected from brackish and fresh water habitats at two locations in the Jaffna peninsula. We also determined whether differences in salinity tolerance between brackish and fresh water-derived *Ae. aegypti* were due to genetic changes by investigating the reversibility of salinity tolerance on transferring brackish water isolates to fresh water and *vice versa* and maintaining such reversal colonies for up to five generations.

## Materials and Methods

### Ethical statement

The owners were informed of the nature of the study and their verbal informed consent obtained when larval collections were carried out in private property. Permission was not required for larval collections in public land as this did not involve endangered species or protected areas. The care and use of mice were according to WHO guidelines (WHO/LAB/88.1) and the protocol for using anaesthetised Balb/c mice for feeding mosquitoes was approved by the Animal Ethics Review Committee of the University of Jaffna (AERC/2014/02).

### Mosquito isolates

**Figure 1:**
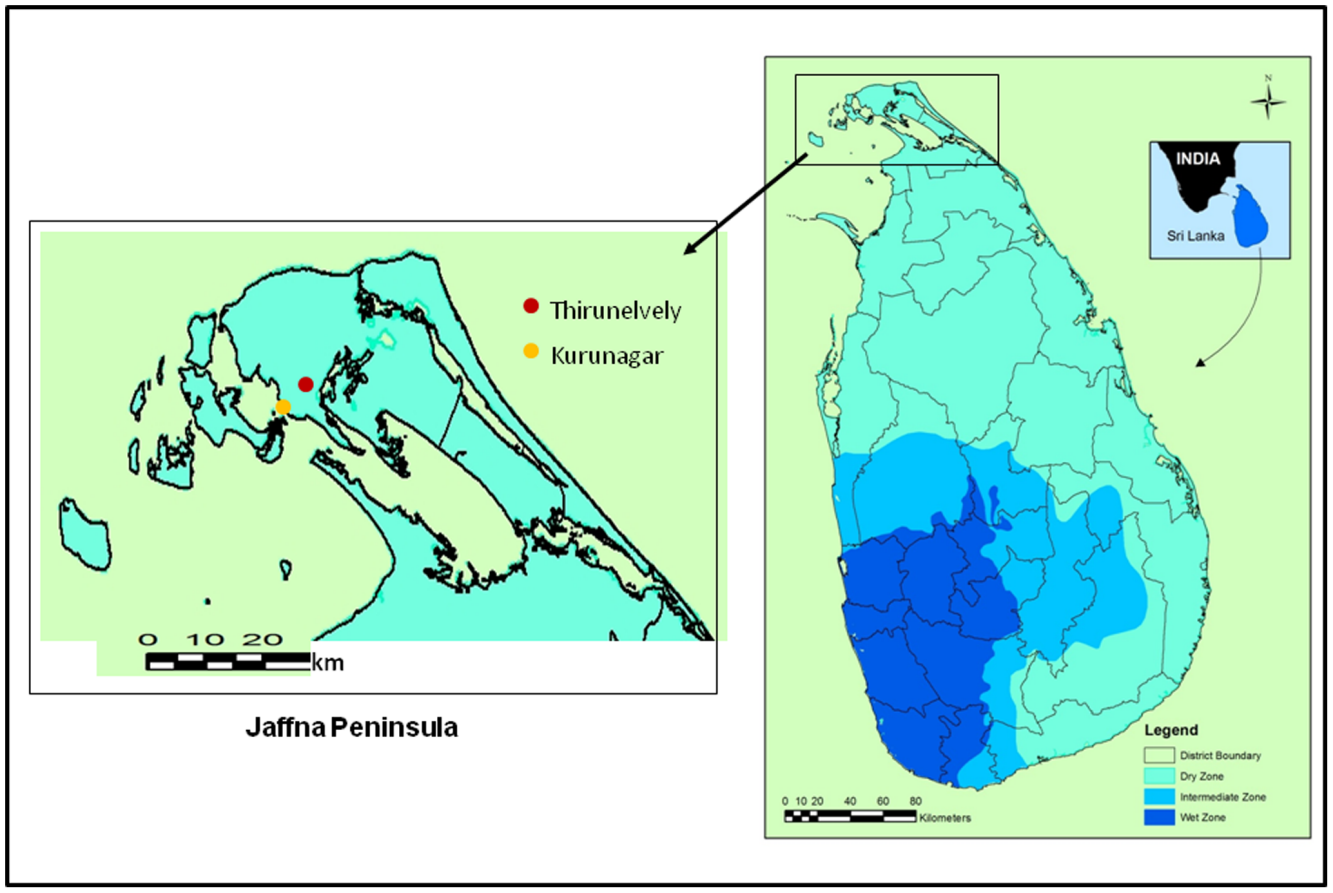
Map of Sri Lanka showing the respective location of brackish and fresh water larval collection sites at Kurunagar and Thirunelvely. The two sites are located in the northern Jaffna peninsula. The map also shows the boundaries of the different administrative districts and the dry, intermediate and wet rainfall zones in the country.

*Ae. aegypti* larvae were collected from brackish water of 2–8 ppt salinity in domestic wells and water tanks and from ovitraps containing brackish water of 10 ppt salinity in the Kurunagar coast of Jaffna city (9°39′N: 80°1′E) in northern Sri Lanka (Figure 1) as previously described [10], [12]. The larvae were maintained at 10 ppt salinity in the insectary of the Department of Zoology at ambient temperature (28±2°C) with fish meal powder provided twice a day as larval food. Emergent adults were used to establish a self-mating colony of *Ae. aegypti* that was maintained in 10 ppt salinity. At the same time, fresh water ovitraps (0 ppt salinity) were used to collect *Ae. aegypti* larvae in Thirunelvely (9°41′N: 80°1′E) in the centre of the Jaffna peninsula (Figure 1), the larvae subsequently maintained in fresh water (0 ppt salinity) and the emergent adults used to establish a self-mating colony of fresh water *Ae. aegypti*. The mosquitoes were fed every three days on Balb/c mouse blood and 10% glucose pledgets were provided at other times. Two separate collections of *Ae. aegypti* were made at Kurunagar and Thirunelvely in October 2012 (onset of the rainy season) and February 2013 (dry season) for establishing brackish and fresh water colonies for the respective duplicate experiments 1 and 2 described below.

For experiments 1 and 2 that investigated the reversibility of salinity tolerance, reversal colonies were independently generated from the previously established brackish and fresh water colonies of *Ae. aegypti*. From the 10 ppt salinity brackish water colony, a reversal colony was established by transferring approximately 200 larvae into fresh water and maintaining subsequent generations in fresh water. Similarly, a brackish water reversal colony was established from the fresh water colony by transferring approximately 200 larvae to brackish water containing 10 ppt salt. After having successfully maintained the reversal colonies for two and five generations, the larval progenies from all four colonies maintained in parallel for each experiment, *viz*. original brackish water colony in 10 ppt, brackish water colony transferred to fresh water (reversal colony), original fresh water colony in 0 ppt and fresh water colony transferred to brackish water at 10 ppt (reversal colony) were used to determine their comparative salinity tolerance.

### Comparative salinity tolerance of brackish and fresh water *Ae. aegypti* and its reversibility

In each of two independent experiments performed in October–November 2012 and February–March 2013, first instar larvae of *Ae. aegypti* from the four colonies were exposed to salinities of 0, 2, 4, 6, 8, 10, 12, 14, 18 and 20 ppt following methods previously described [10]. The required salinities were obtained by adding tap water to seawater. Salinity was measured using a refractor salinometer (Atago, Japan). Twenty larvae in 150 ml capacity plastic containers containing 100 ml of water of specific salinity were maintained at room temperature (28±2°C) until their emergence as adults. Three replicates using 20 larvae each were performed in parallel at each salinity level for the four colonies in both experiments. Plastic lids were used to cover the containers to minimize evaporation. Test media were changed on alternate days. Larvae were fed twice daily with locally available powered fish meal. Mortality of larvae and the numbers of emerging adults were determined.

### Oviposition preference of brackish and fresh water *Ae. aegypti*

Fifty blood-fed three day-old females from the original brackish and fresh water colonies after eight generations in the laboratory were released into separate mosquito cages (one for brackish water and the other for fresh water-derived females). Each cage was provided with three fresh water and three 10 ppt salinity brackish water egg laying surfaces. The numbers of eggs laid on each type of egg-laying surface were determined after three days. The experiment was repeated three times.

### Hatching of *Ae. aegypti* eggs and larval development times in brackish and fresh water

One hundred and fifty eggs from each of the four colonies *viz.* the two original colonies and the two reversal colonies described above (fifth generation of reversal colonies for experiment 1 and second and fifth generations of reversal colonies for experiment 2) were collected and stored at ambient temperature (28±2°C) for 1–2 weeks. They were then flooded as appropriate with 10 ppt brackish water or fresh water to initiate hatching. The numbers of eggs hatching into larvae were determined after 48 hours. Developed larvae were fed twice daily with locally available powered fish meal. The duration of larval development were then determined by counting the numbers of pupae produced at different times. Three replicates were run in parallel for each determination.

### Ability of brackish and fresh water-derived *Ae. aegypti* to interbreed

After eight generations in the laboratory, 50 three and four day-old females from the brackish water-derived *Ae. aegypti* and 50 males from fresh water-derived *Ae. aegypti* colonies were allowed to mate naturally in a mosquito cage under standard insectary conditions (12 hour dark and light; 28±2°C). After 2 days and over-night starvation, they were fed on mouse blood and a fresh water egg-laying surface supplied. Fifty females from the fresh water colony were similarly mated with 50 males from the brackish water colony. The laid eggs were counted and allowed to hatch in fresh water and the emerging F1 male adult progeny from each cross were back-crossed with respective females from the original parent colony to test the viability of F1 males.

### Susceptibility of brackish and fresh water isolates of *Ae. aegypti* to insecticides

Separate collections of larvae from brackish water containers (2–4 ppt salinity) in coastal Kurunagar and fresh water ovitraps (0 ppt salinity) in inland Thirunelvely respectively were made between November 2011 and March 2012 as previously described [10] for testing their susceptibility to insecticides. The first generation of adults emerging from the field-collected larvae were exposed to three insecticides commonly used in the Jaffna peninsula, *viz.* permethrin (0.25%), propoxur (0.1%) and malathion (4%). Susceptibility bioassays were conducted using WHO standard bioassay kits as described previously [23]. Based on availability, 10 to 20 females, aged two to three days, were exposed to insecticide impregnated papers for one hour. Three replicate determinations were made for each insecticide. Papers impregnated with the solvent alone were used as controls. Dead mosquitoes were counted after a recovery period of 24 hours.

### Statistical analysis

The concentration of salt causing 50% mortality in the first instar larvae to adult transition (LC_50_) was determined with 95% confidence limits using the Minitab 14 statistical software (Minitab Inc, PA, USA) as previously described [10]. LC_50_ ratio tests were done to further determine the significance of LC_50_ variations between test populations as described by Wheeler *et al.* [24]. Two-tailed Student's t tests were performed to determine the significance of differences in mean numbers of eggs laid and eggs hatching into larvae, and the mean percentage susceptibility to insecticides using the Minitab statistical software.

## Results

### Salinity tolerance of fresh and brackish water *Ae. aegypti*

**Figure 2:**
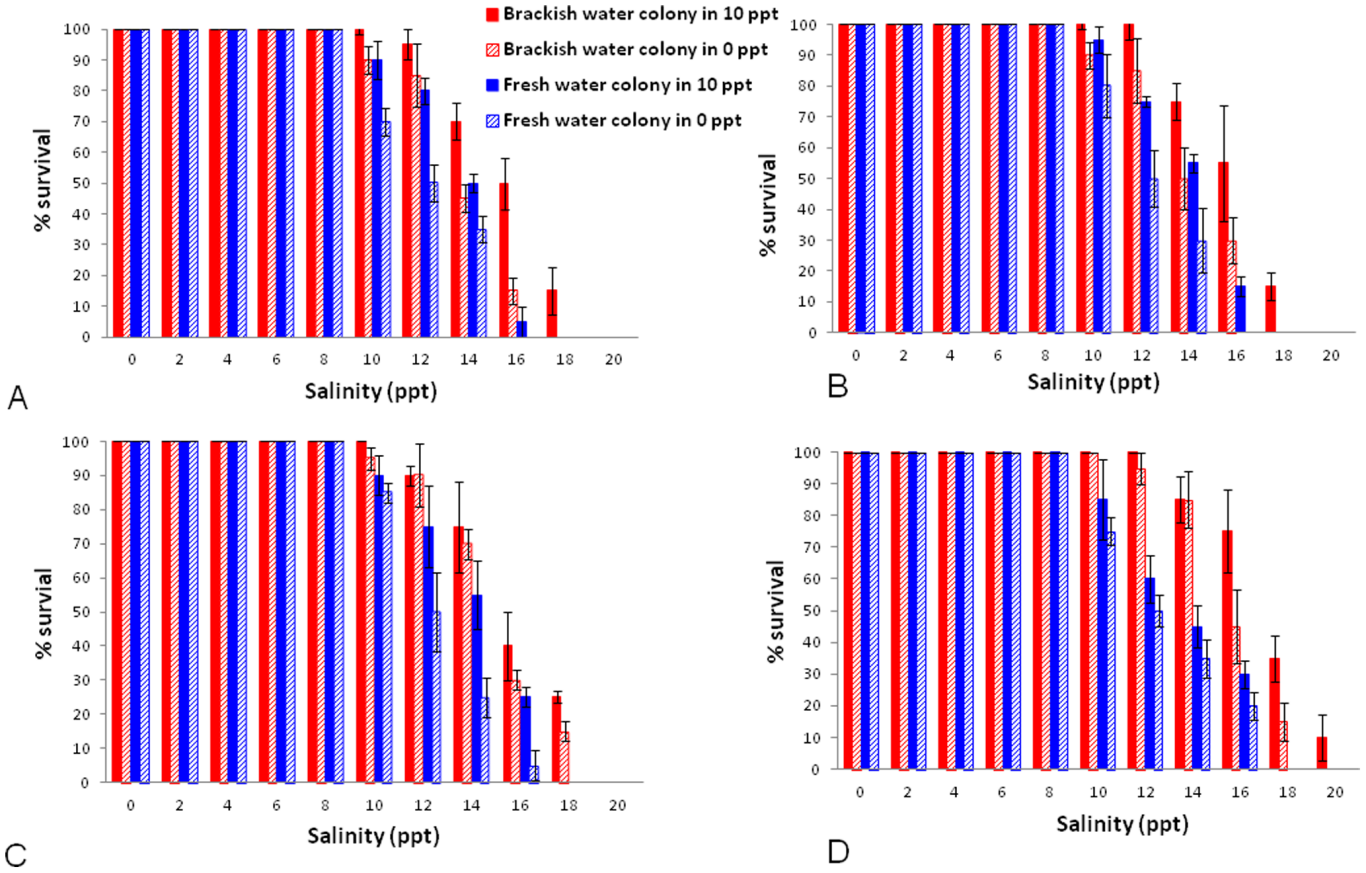
Salinity tolerance of brackish and fresh water-derived *Ae. aegypti* from Kurunagar and Thirunelvely respectively. The mean percent survival of first instar larvae to adulthood at each salinity level together with standard errors of the means of triplicate determinations are shown for the different colonies. Larvae were derived from brackish and fresh water colonies maintained at the original salinity and after reversal of salinity for two and five generations. A. Experiment 1 - second generation; B. Experiment 2 - second generation; C. Experiment 1 - fifth generation; D. Experiment 2 - fifth generation.

The results from the two independent experiments performed at different times on the effect of salinity on the first instar larvae to adult transition in the different colonies are presented in Figure 2. They show that brackish water-derived first instar *Ae. aegypti* larvae maintained at 10 ppt salinity demonstrated 100% survival to adulthood at salinities up to 12 ppt (Figure 2 B&D) while 100% survival in fresh water-derived *Ae. aegypti* maintained in fresh water was only observed up to 8 ppt salinity (Figure 2 A–D).

These findings were also reflected in the maximal salinity tolerance defined as the highest salinity that permitted any larvae to survive to become adults. For example, some larvae from the brackish water colony maintained in 10 ppt salinity survived 20 ppt salt (Figure 2 D) while larvae from the fresh water colony maintained in fresh water could maximally tolerate only 16 ppt salt (Figure 2 C&D).

Furthermore, brackish water-derived *Ae. aegypti* even after five generations of maintenance in fresh water had higher maximal salinity tolerance (18 ppt, Figure 2 C&D) and show 100% survival at higher salinity (10 ppt, Figure 2 D) than fresh water-derived *Ae. aegypti* maintained in fresh water. Similarly, fresh water-derived *Ae. aegypti* after five generations of maintenance in 10 ppt brackish water showed 100% survival at lower salinity (8 ppt Figure 2 C&D) and lower maximal salinity tolerance (16 ppt Figure 2 C&D) than the brackish water colony maintained at 10 ppt salinity throughout.

We also determined the LC_50_ for salinity tolerance as the ppt salt that causes 50% mortality in the transition from first instar larvae to adults for statistical analysis (Table 1). The LC_50_ of the original brackish water colony that continued to be maintained at 10 ppt salinity remained significantly greater than the LC_50_ for the fresh water colony maintained in fresh water for up to five generations, based on non-overlapping 95% confidence intervals in both experiments. This was confirmed by LC_50_ ratio tests [24] at the p<0.001 level of significance (Table S1). Furthermore, the fresh water colony that was transferred to 10 ppt salinity always had lower LC_50_ than the original brackish water colony maintained at 10 ppt salinity at the p<0.005 level of significance (Table S1). These findings show that even after five generations of adaptation to brackish water, the fresh water-derived *Ae. aegypti* never become as salinity tolerant as the brackish water-derived *Ae. aegypti*. Conversely, the brackish water-derived *Ae. aegypti* that were transferred to fresh water remained significantly more salinity tolerant in terms of LC_50_ at the p<0.005 level than the original fresh water colony maintained in fresh water (Table S1). These findings show that even after five generations of adaptation to fresh water, the brackish water-derived *Ae. aegypti* remain more salinity tolerant than fresh water-derived *Ae. aegypti*. The LC_50_ values for brackish water-derived *Ae. aegypti* that were transferred to fresh water were always higher than that of the fresh water colony transferred to 10 ppt brackish water in every comparison but the differences were not statistically significant (Table S1).

**Table 1:** LC_50_ for salinity tolerance of *Aedes aegypti* colonies.

Experiment number and the generation of larvae tested	Colonies			
	Brackish water colony maintained at 10 ppt salinity	Reversal brackish water colony transferred to 0 ppt salinity	Reversal fresh water colony transferred to 10 ppt salinity	Fresh water colony maintained at 0 ppt salinity
Experiment 1, 2^nd^ generation	15.6 (14.9 – 16.3)	13.7 (13.0–14.4)	13.4 (12.8–14.1)	12.1 (11.4–12.8)
Experiment 2, 2^nd^ generation	15.6 (14.9–16.4)	15.0 (14.2–15.8)	13.9 (13.1–14.6)	12.3 (11.6–13.0)
Experiment 1, 5th generation	15.9 (15.2–16.6)	14.1 (13.3–14.8)	13.8 (13.1–14.5)	12.2 (11.5–12.9)
Experiment 2, 5th generation	17.1 (16.4–17.8)	15.8 (15.1–16.5)	15.4 (14.6–16.2)	13.0 (12.2–13.8)

LC_50_ is the salt concentration in parts per thousand (ppt) that results in 50% mortality in the transition from first instar larvae to adults.The 95% confidence intervals of the LC_50_ values are shown in parentheses. The original brackish and fresh water colonies were derived from *Ae. aegypti* collected in Kurunagar and Thirunelvely respectively in the Jaffna peninsula.

The transfer of brackish water-derived *Ae. aegypti* to fresh water always resulted in lower salinity tolerance than the original brackish water colony that was reflected in a reduced LC_50_ that was significant at the p<0.05 level in three out of the four comparisons (Table S1). Significant lowering of LC_50_ was seen even after two generations in fresh water in experiment 1 (Tables 1 and S1). Similarly the transfer of fresh water-derived *Ae. aegypti* to brackish water resulted in the development of greater salinity tolerance that was reflected in higher LC_50_ values than the original fresh water colony that was significant at the p<0.01 level after two and five generations of reversal (Table S1).

The LC_50_ for salinity tolerance in the respective original colonies were not significantly different between experiments 1 and 2 after two generations but diverged for the original brackish water colonies after five generations in the laboratory (Tables 1 and S2). The LC_50_ values between the corresponding reversal colonies in the two experiments also increasingly diverged with increasing number of generations (Tables 1 and S2).

In summary, the results in the two individual experiments show that brackish water-derived *Ae. aegypti* are more salinity tolerant than fresh water-derived *Ae. aegypti* and that this difference was partly reduced when the respective salinities at which they were being maintained were reversed for up to five generations.

### Preferences of brackish and fresh water-derived *Ae. aegypti* to oviposit on brackish and fresh water surfaces

The preferences of blood-fed female *Ae. aegypti* from the two original colonies, maintained in 10 ppt brackish water and 0 ppt fresh water for eight generations after field collection, to oviposit on 10 ppt brackish or fresh water surfaces are shown in Table 2. The results show that female fresh water-isolated *Ae. aegypti* significantly preferred fresh water over brackish water for oviposition. In contrast, females from brackish water-derived *Ae. aegypti* tended to lay more eggs in brackish than fresh water surfaces but the difference was not statistically significant.

**Table 2:** Preference of blood-fed females from brackish and fresh water- derived *Ae. aegypti* for oviposition on brackish and fresh water surfaces.

Experiment	Numbers of eggs laid			
	Brackish water colony (10 ppt)		Fresh water colony (0 ppt)	
	Fresh water surfaces (0 ppt salinity)	Brackish water surfaces (10 ppt salinity)	Fresh water surfaces (0 ppt salinity)	Brackish water surfaces (10 ppt salinity)
Experiment 1	371	466	696	171
Experiment 2	266	406	498	101
Experiment 3	486	485	627	118
Mean ± sd	374±110	452±41	607±101	130±37
Student's t test value T and probability P	T = 1.15, P = 0.31		T = 7.68, P = 0.001	

The results are the mean number of eggs laid in the two types of surfaces ± standard deviation in the three separate experiments. A two-tailed unpaired Student's t test was performed to determine the probability of significant differences in the mean number of eggs laid by females of each colony on brackish and fresh water surfaces. The brackish and fresh water colonies were derived from *Ae. aegypti* collected in Kurunagar and Thirunelvely respectively in the Jaffna peninsula.

### Hatching of *Ae. aegypti* eggs in brackish and fresh water

The numbers of eggs hatching from an initial number of 150 eggs after 48 h from the original brackish water colony, the original fresh water colony, the reversal brackish water colony transferred to fresh water and the reversal fresh water colony transferred to brackish water in their respective salinities are shown in Table 3. Egg hatching was quantitatively determined for the 5^th^ generation of the colonies in experiment 1 and for both 2^nd^ and 5^th^ generations in experiment 2. The numbers of eggs hatching in fresh water were significantly higher than in 10 ppt brackish water regardless of the colony from which the eggs were derived. The original brackish water-derived *Ae. aegypti* colony tended to have more eggs hatching in 10 ppt brackish water than the reversal colony of fresh water-derived *Ae. aegypti* transferred to 10 ppt salt brackish water, but the differences were not statistically significant.

**Table 3:** Hatching of *Ae. aegypti* eggs in brackish and fresh water.

Experiment	Number of eggs out of 150 from different colonies hatching into larvae at 48 h			
	Original fresh water colony at 0 ppt salinity	Reversal fresh water colony at 10 ppt salinity	Reversal brackish water colony at 0 ppt salinity	Original brackish water colony at 10 ppt salinity
5th generation Experiment 1	127±8	63±10*	125±9	86±13*
2^nd^ generation Experiment 2	128±4	61±17*	123±11	74±23*^e^
5th generation Experiment 2	123±7	73±9*	121±8	74±12*

Results are the mean numbers of eggs from three replicate experiments ± standard deviation that hatched into larvae at 48 h from 150 original eggs. Unpaired Student's t tests were performed to determine the significance of differences in the mean numbers of eggs hatching in brackish and fresh water for the corresponding original and reversal colonies (columns 1 *vs* 2 and 3 *vs* 4 respectively) in the three different experiments. The asterisks indicate that in every such comparison significantly more eggs hatched in fresh water compared to brackish water at p<0.05. Corresponding comparisons of the pairs of means in columns 1 *vs* 3 and 2 *vs* 4 were not statistically significant with p>0.05. The original brackish and fresh water colonies were derived from *Ae. aegypti* collected in Kurunagar and Thirunelvely respectively in the Jaffna peninsula.

### Larval development times of *Ae. aegypti* in brackish and fresh water

**Figure 3:**
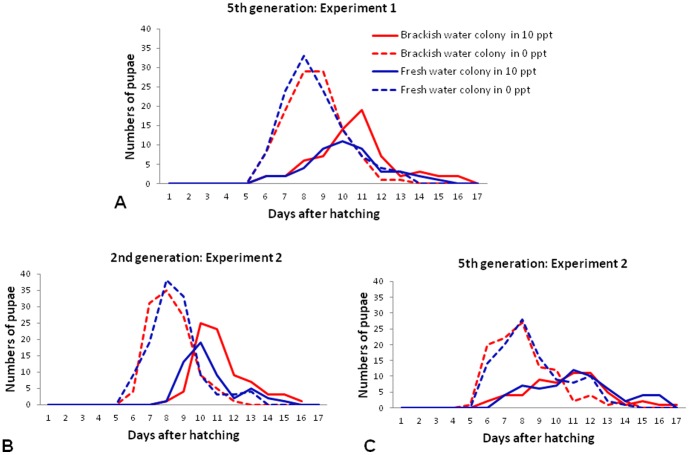
Larval development times of *Ae. aegypti* in fresh and 10 ppt salt brackish water. The results show the mean numbers of *Ae. aegypti* pupae emerging on different days after hatching of eggs derived from the brackish and fresh water colonies maintained at the original salinity and after reversal of salinity for up to five generations. The results are the means of three replicate determinations. A. Experiment 1 with fifth generation larvae, B. Experiment 2 with second generation larvae, C. Experiment 2 with fifth generation larvae. The original brackish and fresh water colonies were derived from *Ae. aegypti* collected in Kurunagar and Thirunelvely respectively in the Jaffna peninsula.

Figure 3 shows the time taken for larvae to develop into pupae in brackish and fresh water in the experiments. The emergence of pupae at different times after hatching was quantitatively determined for the 5^th^ generation of the colonies in experiment 1 and for both 2^nd^ and 5^th^ generations in experiment 2. The results show that larval development occurred more slowly in brackish water of 10 ppt salinity than fresh water, regardless of the brackish or fresh water origin of the colonies from which the eggs were derived. Larval development in fresh water showed a sharper peak of pupation at days seven to nine than in brackish water where there was a more prolonged emergence of pupae with broader peaks at days ten to twelve. Delayed larval development in 10 ppt salinity brackish water was also reflected in the median time to pupation. For the 5^th^ generation in experiment 1, the median times to pupation were 9.5, 10.5, 9.5 and 11.0 days after hatching for the original fresh water colony maintained in fresh water, the reversal fresh water colony transferred to 10 ppt salinity, the reversal brackish water colony transferred to fresh water and the original brackish water colony maintained in 10 ppt salinity respectively. The corresponding median times to pupation for the four colonies were 9.5, 11.5, 9.0, 12.0 and 10.0, 11.5, 9.5 and 11.5 days for the 2^nd^ and 5^th^ generations respectively in experiment 2.

### Interbreeding between brackish and fresh water-derived *Ae. aegypti*

Because of the differences between the brackish and fresh water-derived *Ae. aegypti*, we investigated the possible existence of a reproductive barrier between the two populations. In the mating experiment between brackish water-derived females and fresh water-derived males, 657 eggs were collected and of these 73.5% hatched into larvae. In the back cross between these F1 males and parental brackish water-derived females, 544 egg were produced and these showed 74.6% hatchability. The mating of brackish water-derived males and fresh water-derived females produced 749 eggs with 75.8% hatchability, while 923 eggs were produced in the backcross between these F1 males and parental fresh water-derived females with 77.3% hatchability. The results show that there are no reproductive barriers between the coastal brackish water and inland fresh water populations of *Ae. aegypti* derived from Kurunagar and Thirunelvely respectively.

### Differential susceptibility to insecticides in brackish and fresh water *Ae. aegypti* isolates

The insecticide bioassays show that *Ae. aegypti* collected from fresh water ovitraps in inland Thirunelvely were significantly more resistant to malathion than *Ae. aegypti* collected from brackish water habitats in coastal Kurunagar (Table 4). The differences between the brackish and fresh water-derived *Ae. aegypti* were not statistically significant for propoxur and permethrin. Both brackish and fresh water-derived *Ae. aegypti* were relatively resistant to the carbamate insecticide propoxur.

**Table 4:** Resistance of field-collected brackish and fresh water populations of *Ae. aegypti* from Kurunagar and Thirunelvely respectively to common insecticides.

Insecticide	% Resistant		Student's t test value T and Probability P
	Coastal brackish water population from Kurunagar	Inland fresh water population from Thirunelvely	
Permethrin (0.25%)	19±6	30±9	T = 1.31, P = 0.32
Propoxur (0.1%)	74±13	78±17	T = 0.24, P = 0.83
Malathion (4%)	34±8	79±14	T = 6.24, P = 0.02

The results are the means ± standard deviation of three replicate determinations. A two-tailed Student's t test for matched samples was performed to determine the probability of significant differences in the mean resistance of brackish and fresh water populations to the three insecticides.

## Discussion

Wallis showed in 1954 that long-established laboratory colonies of *Ae. aegypti* prefer to oviposit in fresh water or 2.5 ppt salinity and that oviposition is inhibited at higher salinities [25]. Wallis also found that the chemosensors for sodium chloride relevant to oviposition were located in all the tarsomeres in *Ae. aegypti* legs [25]. Previous studies in Sri Lanka showed that *Ae. aegypti* can oviposit in water of up to 18 ppt salt in containers and wells in the peri-urban environment as well as ovitraps placed in the field [10]–[12]. However egg laying in field ovitraps decreased with increasing salinity [10]. The present findings show that *Ae. aegypti* derived from fresh water ovitraps in Thirunelvely and maintained in fresh water for eight generations clearly prefer fresh water to 10 ppt brackish water for oviposition. In contrast, *Ae. aegypti* collected from brackish water at Kurunagar and maintained at 10 ppt salinity for eight generations did not significantly differentiate between 10 ppt brackish and fresh water surfaces for oviposition. The results suggest that there has been an adaptation to enable greater oviposition in brackish water in brackish water-derived *Ae. aegypti* that could involve alterations in the physiological mechanisms for sensing and responding to sodium chloride. It is possible that a greater adaptation to salinity in oviposition may be demonstrable at salinities <10 ppt in brackish water-derived *Ae. aegypti*. This merits further investigation as there are many brackish water habitats with <10 ppt salinity where *Ae. aegypti* larvae are found in Kurunagar [10], [12]

The present findings also show that the hatching of eggs from the brackish or fresh water-derived *Ae. aegypti* is inhibited by 10 ppt salt demonstrating that there has been no significant adaptation to 10 ppt salinity in brackish water-derived *Ae. aegypti* in this respect even after five generations of maintenance at 10 ppt salt in the laboratory. It is possible, as discussed above for oviposition, that significant adaptation to salinity in egg hatching may be demonstrable at salinities <10 ppt. Our observations are compatible with recent findings in Florida, USA which showed that high salinities inhibited egg hatching in *Ae. aegypti* [18].

Studies in the USA have shown that larval development times are prolonged and pupal mass reduced at high salinities in *Ae. aegypti* [18], [26]. *Ae. aegypti* larvae from long-established laboratory colonies were able to survive at salinity up to 17.5 ppt [26] while larvae from short – term colonies showed positive growth at 10 ppt salinity [18]. Our findings show that larval development times are prolonged at 10 ppt salinity in both brackish and fresh water-derived *Ae. aegypti*. This shows that brackish water-derived *Ae. aegypti* from Kurunagar have not detectably adapted to salinity in this respect, and this remains so even after five generation of maintenance at 10 ppt salinity in the laboratory. As discussed above for oviposition and egg hatching, it is possible that evidence of adaptation in larval development times may be demonstrable at <10 ppt salinity, and this merits further investigation.

Our previous study suggested that *Ae. aegypti* isolated in Thirunelvely in the Jaffna peninsula differed in salinity tolerance from *Ae. aegypti* isolated in Batticaloa in the eastern province of mainland Sri Lanka [10]. The difference was attributed to the genetic variation caused by adaptation to the generally higher ground water salinity in the Jaffna peninsula [10]. We therefore looked for differences in salinity tolerance between *Ae. aegypti* collected from coastal brackish water and inland fresh water habitats in Kurunagar and Thirunelvely respectively within the Jaffna peninsula. Our findings show that coastal brackish water-derived *Ae. aegypti* from Kurunagar are more tolerant of salinity than inland fresh water-derived *Ae. aegypti* from Thirunelvely in the Jaffna peninsula. Our findings also suggest that the difference in salinity tolerance between coastal and inland *Ae. aegypti* isolates from Kurunagar and Thirunelvely respectively are only partially reduced after five generations of reversal of salinity in the laboratory. Biological changes associated with adaptation to brackish water in *Ae. aegypti* that are not readily reversible are likely to be caused by underlying genetic and/or epigenetic changes that can only be characterised through appropriate genetic and molecular biological studies. However, the present data also show that brackish water-derived *Ae. aegypti* from Kurunagar have not yet undergone biological changes that overcome less efficient hatching of eggs and slower larval development in 10 ppt brackish water.

It is possible that one or more of the physiological and structural changes associated with salinity tolerance previously demonstrated in *Ae. aegypti* [17] and other mosquito species [19]–[22] contribute to the greater salinity tolerance of brackish water-derived *Ae. aegypti* from coastal Kurunagar. The underlying genetic and physiological mechanisms need to be elucidated in more detailed investigations.

Collection of larvae for experiments 1 and 2 and the respective experiments were performed four months apart. While there were no significant differences in the LC_50_ after two generations between the respective original colonies in the two experiments, the LC_50_ between corresponding colonies in the two experiments tended to diverge with increasing number of generations and reversal of salinity. Differential responses in the complex biological mechanisms underlying responses to salinity changes and laboratory colonisation may be responsible for these differences between the two experiments undertaken four months apart. However, it is clear that within each experiment, brackish water-derived *Ae. aegypti* always showed significantly higher salinity tolerance than fresh water-derived *Ae. aegypti* and that this difference was only partly reduced by reversing the corresponding salinities for five generations.

Our findings show that brackish water-derived *Ae. aegypti* from Kurunagar remain relatively susceptible to malathion (an organophosphate insecticide) and permethrin (a pyrethroid). These and related insecticides of the two classes may therefore be useful for controlling brackish water-derived coastal populations of *Ae. aegypti* in Kurunagar. The differential susceptibility to malathion also suggests that there are genetic differences between brackish water-derived *Ae. aegypti* from coastal Kurunagar and fresh water-derived *Ae. aegypti* from inland Thirunelvely in the Jaffna peninsula. Malathion was widely used for indoor residual spraying of houses to control malaria vectors in the Jaffna peninsula from the mid 1970s until early 2000s, when it was replaced with pyrethroids. The organophosphate Temephos is presently used as a larvicide for *Aedes* dengue vectors solely in fresh water habitats. Other organophosphate insecticides continue to be used in inland areas of the peninsula for controlling agricultural pests. Coastal isolates of *Anopheles subpictus* in Sri Lanka were recently shown to be less resistant to malathion and pyrethroids than inland isolates, and this was attributed to the widespread use of insecticides in inland areas [23]. It is therefore possible that the use of organophosphate insecticides in predominantly inland locations in the Jaffna peninsula has led to inland fresh water *Ae. aegypti* populations developing greater resistance to the malathion, which can be mediated by mutations in the target acetylcholinesterase and changes in glutathione S-transferase and carboxylesterases that metabolise malathion [23].

However, further sampling studies are needed to determine how representative the phenotypic differences observed between brackish water-derived *Ae. aegypti* from Kurunagar and fresh water-derived *Ae. aegypti* from Thirunelvely in the Jaffna peninsula are of brackish and fresh water-derived *Ae. aegypti* populations elsewhere in Sri Lanka or other countries. There is also a need to determine the relative vectorial capacities of brackish and fresh water *Ae. aegypti* populations at the two locations in the Jaffna peninsula and elsewhere.

Genetic differences between *Ae. aegypti* populations in Venezuela [27] and North Queensland [28] have been documented using polymorphic DNA markers. Similarly microsatellite analysis has shown habitat-based population structuring in the closely related *Ae. albopictus* over short distances in Reunion island in the South Indian Ocean [29]. Coastal Kurunagar (the origin of brackish water *Ae. aegypti* used in the study) and inland Thirunelvely (the origin of fresh water *Ae. aegypti*) in the Jaffna peninsula are 5 km apart. *Ae. aegypti* are short distance migrants that normally lay eggs within 1 km from the site of a blood meal [30], [31]. The present findings are therefore compatible with the possibility that genetic differences have developed, despite the relatively short distance involved, due to restricted gene flow between coastal brackish water populations of *Ae. aegypti* from Kurunagar and inland fresh water populations from Thirunelvely. An analogous situation has been observed in South-West Australia where larvae of coastal marsh populations of *Aedes camptorhynchus* (a vector of Ross River virus) tolerate greater salinity (52 ppt, i.e. hypersalinity) than inland populations (30 ppt, i.e. approaching the average salinity of sea water), probably due to genetic changes in osmoregulatory mechanisms [32]. However, the present data show that likely genetic differences between the coastal and inland isolates of *Ae. aegypti* from Kurunagar and Thirunelvely respectively in the Jaffna peninsula have not yet become a barrier to reproduction. There is a possibility that brackish water *Ae. aegypti* can develop into a separate species given sufficient time and physical isolation from the original fresh water species. The evolution of salinity-tolerant *Anopheles melas* and *Anopheles merus* in Africa within the *Anopheles gambiae* complex provides a precedent for such a process [33].

It is not presently clear to what extent the development of *Ae. aegypti* in brackish water in Kurunagar has been driven by selective pressure exerted by dengue control measures applied to fresh water habitats or the opportunistic exploitation of a recent proliferation of anthropogenic ecological niches near human dwellings in coastal areas. It is likely that both are contributing factors. However the evidence suggests that the utilisation of brackish water habitats by *Ae. aegypti* may be associated at the present time with less efficient hatching of eggs and slower larval development at higher salinities that are presumably balanced by the other advantages.

Analysis of the temporal relationship between dengue incidence and rainfall suggests that monsoonal rains are important drivers of dengue transmission in the Jaffna district [14]. Coastal Kurunagar has a high incidence of dengue [10]. It is therefore possible that coastal brackish water *Aedes* vectors in the Jaffna peninsula constitute an unappreciated, perennial source of dengue transmission that promotes increased transmission following the monsoon. Similar considerations may apply to coastal locations elsewhere in Sri Lanka and other dengue-endemic countries.

The present findings further support assertions [10]–[16], [18] that existing guidelines on dengue control [5] need to be extended to target brackish water habitats of *Ae. aegypti* and *Ae. albopictus* in the urban and peri-urban environment. They also highlight a global need for more research into the genetic and physiological basis for salinity adaptation in vectors and the role of salinity tolerant vectors in disease transmission, and the formulation of appropriate mitigating measures in a future context of rising sea levels [14]–[16].

## Supporting Information

Table S1 **Statistical comparison of LC_50_ values for salinity tolerance between the different original and reversal colonies of** ***Aedes aegypti*** **after two and five generations in the laboratory in Experiments 1 and 2.**
(DOC)

Table S2 **Statistical comparison of LC_50_ values for salinity tolerance between corresponding colonies of** ***Aedes aegypti*** **from the two different collections used in Experiments 1 and 2.**
(DOC)
