# Supplementary material for: Biological Differences between Brackish and Fresh Water-Derived Aedes aegypti from Two Locations in the Jaffna Peninsula of Sri Lanka and the Implications for Arboviral Disease Transmission
Source: PLoS One. 2014 Aug 29;9(8):e104977. doi: 10.1371/journal.pone.0104977 (PMC4149417; doi:10.1371/journal.pone.0104977)
Supplement: Table S1 — Statistical comparison of LC50 values for salinity tolerance between the different original and reversal colonies of Aedes aegypti after two and five generations in the laboratory in Experiments 1 and 2. (DOC) [file pone.0104977.s001.doc]

**Table S1. Statistical comparison of LC_50_ values for salinity tolerance between the different original and reversal colonies of *Aedes aegypti* after two and five generations in the laboratory in Experiments 1 and 2**

| **Colonies** | **Experiment & Generation** | **LC_50_ (CI)** | **LC_50_ ratio test statistic** | **Standard error** | **Test statistic Z** | **p** |
| --- | --- | --- | --- | --- | --- | --- |
| Brackish water colony in 10 ppt *vs* Fresh water colony in 10 ppt | Exp-1, 2^nd^ *vs* Exp-1, 2^nd^ | 15.6 (14.9-16.3) 13.4 (12.8-14.1) | 0.149 | 0.033 | 4.4 | 0.000 |
| Brackish water colony in 10 ppt *vs* Fresh water colony in 10 ppt | Exp-1, 5^th^ *vs* Exp-1, 5^th^ | 15.9 (15.2-16.6) 13.8 (13.1-14.5) | 0.264 | 0.230 | 1.2 | 0.000 |
| Brackish water colony in 10 ppt *vs* Fresh water colony in 10 ppt | Exp-2, 2^nd^ *vs* Exp-2, 2^nd^ | 15.6 (14.9-16.4) 13.9 (13.1-14.6) | 0.119 | 0.036 | 3.3 | 0.000 |
| Brackish water colony in 10 ppt *vs* Fresh water colony in 10 ppt | Exp-2, 5^th^ *vs* Exp-2, 5^th^ | 17.1 (16.4-17.8) 15.4 (14.6-16.2) | 0.103 | 0.038 | 2.7 | 0.006 |
| Brackish water colony in 10 ppt *vs* Fresh water colony in 0 ppt | Exp-1, 2^nd^ *vs* Exp-1, 2^nd^ | 15.6 (14.9-16.3) 12.1 (11.4-12.8) | 0.254 | 0.037 | 6.8 | 0.000 |
| Brackish water colony in 10 ppt *vs* Fresh water colony in 0 ppt | Exp-1, 5^th^ *vs* Exp-1, 5^th^ | 15.9 (15.2-16.6) 12.2 (11.5-12.9) | 0.264 | 0.034 | 7.7 | 0.000 |
| Brackish water colony in 10 ppt *vs* Fresh water colony in 0 ppt | Exp-2, 2^nd^ *vs* Exp-2, 2^nd^ | 15.6 (14.9-16.4)  12.3 (11.6-13.0) | 0.236 | 0.036 | 6.4 | 0.000 |
| Brackish water colony in 10 ppt *vs* Fresh water colony in 0 ppt | Exp-2, 5^th^ *vs* Exp-2, 5^th^ | 17.1 (16.4-17.8) 13.0 (12.2-13.8) | 0.283 | 0.037 | 7.5 | 0.000 |
| Brackish water colony in 0 ppt *vs* Fresh water colony in 10 ppt | Exp-1, 2^nd^ *vs* Exp-1, 2^nd^ | 13.7 (13.0-14.4) 13.4 (12.8-14.1) | 0.016 | 0.035 | 0.5 | 0.641 |
| Brackish water colony in 0 ppt *vs* Fresh water colony in 10 ppt | Exp-1, 5^th^ *vs* Exp-1, 5^th^ | 14.1 (13.3-14.8) 13.8 (13.1-14.5) | 0.021 | 0.036 | 0.6 | 0.563 |
| Brackish water colony in 0 ppt *vs* Fresh water colony in 10 ppt | Exp-2, 2^nd^ vs Exp-2, 2^nd^ | 15.0 (14.2-15.8) 13.9 (13.1-14.6) | 0.078 | 0.282 | 0.3 | 0.781 |
| Brackish water colony in 0 ppt *vs* Fresh water colony in 10 ppt | Exp-2, 5^th^ *vs* Exp-2, 5^th^ | 15.8 (15.1-16.5) 15.4 (14.6-16.2) | 0.017 | 0.042 | 0.4 | 0.681 |
| Brackish water colony in 0 ppt *vs* Fresh water colony in 0 ppt | Exp-1, 2^nd^ *vs* Exp-1, 2^nd^ | 13.7 (13.0-14.4) 12.1 (11.4-12.8) | 0.121 | 0.039 | 3.1 | 0.001 |
| Brackish water colony in 0 ppt *vs* Fresh water colony in 0 ppt | Exp-1, 5^th^ *vs* Exp-1, 5^th^ | 14.1 (13.3-14.8) 12.2 (11.5-12.9) | 0.141 | 0.037 | 3.7 | 0.000 |
| Brackish water colony in 0 ppt *vs* Fresh water colony in 0 ppt | Exp-2, 2^nd^ *vs* Exp-2, 2^nd^ | 15.0 (14.2-15.8) 12.3 (11.6-13.0) | 0.195 | 0.282 | 0.7 | 0.000 |
| Brackish water colony in 0 ppt *vs* Fresh water colony in 0 ppt | Exp-2, 5^th^ *vs* Exp-2, 5^th^ | 15.8 (15.1-16.5)  13.0 (12.2-13.8) | 0.197 | 0.041 | 4.7 | 0.000 |
| Brackish water colony in 10 ppt *vs* Brackish water colony in 0 ppt | Exp-1, 2^nd^ *vs* Exp-1, 2^nd^ | 15.6 (14.9-16.3)  13.7 (13.0-14.4) | 0.132 | 0.034 | 3.9 | 0.000 |
| Brackish water colony in 10 ppt *vs* Brackish water colony in 0 ppt | Exp-1, 5^th^ *vs* Exp-1, 5^th^ | 15.9 (15.2-16.6)  14.1 (13.3-14.8) | 0.121 | 0.033 | 3.6 | 0.000 |
| Brackish water colony in 10 ppt *vs* Brackish water colony in 0 ppt | Exp-2, 2^nd^ *vs* Exp-2, 2^nd^ | 15.6 (14.9-16.4)  15.0 (14.2-15.8) | 0.034 | 0.184 | 0.2 | 0.857 |
| Brackish water colony in 10 ppt *vs* Brackish water colony in 0 ppt | Exp-2, 5th *vs* Exp-2, 5^th^ | 17.1 (16.4-17.8)  15.8 (15.1-16.5) | 0.086 | 0.035 | 2.4 | 0.016 |
| Fresh water colony in 10 ppt *vs* Fresh water colony in 0 ppt | Exp-1, 2^nd^ *vs* Exp-1, 2^nd^ | 13.4 (12.8-14.1)  12.1 (11.4-12.8) | 0.105 | 0.038 | 2.7 | 0.006 |
| Fresh water colony in 10 ppt v*s* Fresh water colony in 0 ppt | Exp-1, 5^th^  *vs* Exp-1, 5^th^ | 13.8 (13.1-14.5)  12.2 (11.5-12.9) | 0.117 | 0.037 | 3.2 | 0.001 |
| Fresh water colony in 10 ppt *vs* Fresh water colony in 0 ppt | Exp-2, 2^nd^ *vs* Exp-2, 2^nd^ | 13.9 (13.1-14.6)  12.3 (11.6-13.0) | 0.117 | 0.038 | 3.0 | 0.002 |
| Fresh water colony in 10 ppt *vs* Fresh water colony in 0 ppt | Exp-2, 5^th^ *vs* Exp-2, 5^th^ | 15.4 (14.6-16.2) 13.0 (12.2-13.8) | 0.179 | 0.043 | 4.1 | 0.000 |

LC_50_ is the salt concentration in parts per thousand (ppt) that results in 50% mortality in the transition from first instar larvae to adults. CI - the 95% confidence intervals of the LC_50_ values in parentheses: p – probability. The statistical comparisons were done according to the LC_50_ ratio test described by Wheeler *et al.* [25]. The original brackish and fresh water colonies were derived from *Ae. aegypti* collected in Kurunagar and Thirunelvely respectively in the Jaffna peninsula.
