# Supplementary material for: Biological Differences between Brackish and Fresh Water-Derived Aedes aegypti from Two Locations in the Jaffna Peninsula of Sri Lanka and the Implications for Arboviral Disease Transmission
Source: PLoS One. 2014 Aug 29;9(8):e104977. doi: 10.1371/journal.pone.0104977 (PMC4149417; doi:10.1371/journal.pone.0104977)
Supplement: Table S2 — Statistical comparison of LC50 values for salinity tolerance between corresponding colonies of Aedes aegypti from the two different collections used in Experiments 1 and 2. (DOC) [file pone.0104977.s002.doc]

**Table S2. Statistical comparison of LC_50_ values for salinity tolerance between corresponding colonies of *Aedes aegypti* from the two different collections used in Experiments 1 and 2**

| **Colonies** | **Experiment & Generation** | **LC_50_ (CI)** | **LC50 ratio test statistic** | **Standard error** | **Test statistic Z** | **p** |
| --- | --- | --- | --- | --- | --- | --- |
| Brackish water colony in 10 ppt | Exp-1, 2^nd^ *vs* Exp-2, 2^nd^ | 15.6 (14.9-16.3)  15.6 (14.9-16.4) | 0.002 | 0.033 | 0.1 | 0.948 |
| Brackish water colony in 0 ppt | Exp-1, 2^nd^ *vs* Exp-2, 2^nd^ | 13.7 (13.0-14.4)  15.0 (14.2-15.8) | 0.095 | 0.035 | 2.7 | 0.006 |
| Fresh water colony in 10 ppt | Exp-1, 2^nd^ *vs* Exp-2, 2^nd^ | 13.4 (12.8-14.1)  13.9 (13.1-14.6) | 0.030 | 0.036 | 0.8 | 0.400 |
| Fresh water colony in 0 ppt | Exp-1, 2^nd^ *vs* Exp-2, 2^nd^ | 12.1 (11.4-12.8)  12.3 (11.6-13.0) | 0.018 | 0.040 | 0.5 | 0.645 |
| Brackish water colony in 10 ppt | Exp-1, 5^th^ *vs* Exp-2, 5^th^ | 15.9 (15.2-16.6)  17.1 (16.4-17.8) | 0.074 | 0.029 | 2.4 | 0.013 |
| Brackish water colony in 0 ppt | Exp-1, 5^th^ *vs* Exp-2, 5^th^ | 14.1 (13.3-14.8)  15.8 (15.1-16.5) | 0.109 | 0.038 | 2.8 | 0.004 |
| Fresh water colony in 10 ppt | Exp-1, 5^th^ *vs* Exp-2, 5^th^ | 13.8 (13.1-14.5)  15.4 (14.6-16.2) | 0.116 | 0.039 | 2.9 | 0.003 |
| Fresh water colony in 0 ppt | Exp-1, 5th *vs* Exp-2, 5^th^ | 12.2 (11.5-12.9)  13.0 (12.2-13.8) | 0.054 | 0.040 | 1.3 | 0.190 |

LC_50_ is the salt concentration in parts per thousand (ppt) that results in 50% mortality in the transition from first instar larvae to adults. CI - the 95% confidence intervals of the LC_50_ values in parentheses: p – probability. The statistical comparisons were done according to the LC_50_ ratio test described by Wheeler *et al.* [25]. The original brackish and fresh water colonies were derived from *Ae. aegypti* collected in Kurunagar and Thirunelvely respectively in the Jaffna peninsula.
